# Renin-Angiotensin-Aldosterone System Blockade in Diabetic Nephropathy. Present Evidences

**DOI:** 10.3390/jcm4111908

**Published:** 2015-11-09

**Authors:** Luz Lozano-Maneiro, Adriana Puente-García

**Affiliations:** Division of Nephrology, Department of Internal Medicine, Fuenlabrada University Hospital, Rey Juan Carlos University School of Medicine, Camino del Molino, 2, 28942 Fuenlabrada, Madrid, Spain; E-Mail: apuente@doctor.com

**Keywords:** renin-angiotensin-aldosterone system blockade, combined RAAS blockade, dual RAAS blockade, diabetic nephropathy, diabetic kidney disease, ACE inhibitors, angiotensin receptor blockers, direct renin inhibitors, mineralocorticoid receptor antagonists, aldosterone antagonist

## Abstract

Diabetic Kidney Disease (DKD) is the leading cause of chronic kidney disease in developed countries and its prevalence has increased dramatically in the past few decades. These patients are at an increased risk for premature death, cardiovascular disease, and other severe illnesses that result in frequent hospitalizations and increased health-care utilization. Although much progress has been made in slowing the progression of diabetic nephropathy, renal dysfunction and the development of end-stage renal disease remain major concerns in diabetes. Dysregulation of the renin-angiotensin-aldosterone system (RAAS) results in progressive renal damage. RAAS blockade is the cornerstone of treatment of DKD, with proven efficacy in many arenas. The theoretically-attractive option of combining these medications that target different points in the pathway, potentially offering a more complete RAAS blockade, has also been tested in clinical trials, but long-term outcomes were disappointing. This review examines the “state of play” for RAAS blockade in DKD, dual blockade of various combinations, and a perspective on its benefits and potential risks.

## 1. A Brief Historic Report: Discovery and Development of Renin-Angiotensin-Aldosterone System (RAAS) and Their Blockers

In 1563, the earliest recorded mention of the adrenal gland is Bartolomeo Eustacchio’s copper-etched depiction of “glandulae Renibus incumentes” [1].

In 1898, Robert Tigerstedt, a finnish medical scientist and physiologist, and his student, Per Bergman, made extracts of rabbit kidney, injected them into rabbits, and observed that even a very small amount of the extract increased blood pressure. Their results suggested the kidneys produced a protein, which they named renin, which caused a rise in blood pressure.

In 1939, renin was found not to cause the rise in blood pressure by Irvine Page, but was an enzyme which catalyzed the formation of the substances that were responsible, namely “angiotonin or hypertenisin” (angiotensin), a pleiotropic hormone with several targets, including the kidneys, blood vessels, and nervous system.

In 1953, aldosterone (“electrocortin”) was first isolated by Simpson and Tait.

History of discovery and development of RAAS and their blockers is summarized in Table 1 [2,3,4].

**Table 1:** History of discovery and development of RAAS and their blockers [2,3,4].

Year			Introduced
1563	Bartolomeo Eustacchio	“glandulae Renibus incumentes” (adrenal gland)	
1898	Tigerstedt and Bergman	renin	
1939	Irvine Page	angiotonin or hypertenisin (angiotensin)	
1953	Simpson and Tait	“electrocortin” (aldosterone)	
1957	Gantt and Dyniewicz	spironolactone	1960
1956	Leonard T. Skeggs	angiotensin-converting enzyme (ACE)	
1965	Ferreira	bradykinin-potentiating factor (BPF)	
1970	Ng and Vane	angiotensin-converting enzyme inhibition	
1975	Squibb	captopril	1977
1979	Merck	enalapril	1981
1993	Takeda-Merck	losartan	1995
1997	Pfizer	eplerenone	2002
2005	Novartis-Speedel	aliskiren	2007

In a patient, it is now possible to inhibit renin directly with DRIs, to block the conversion of angiotensin I to angiotensin II with ACEIs, to block the major downstream targets of Angiotensin II with ARBs, and to blocks the action of aldosterone with AAs.

## 2. Use of RAAS Blockade in Diabetic Kidney Disease (DKD): Past and Present Evidences

Dysregulation of the RAAS has a critical role in the pathogenesis of CKD. There is an increase of glomerular capillary pressure due to continuous stimulation from the RAAS, which enhances oxidative stress and stimulates pro-inflammatory pathways. These mechanisms promote glomerular hypertrophy, which is present at an early stage of DKD and is the beginning of profibrotic cascade [5].

ACEIs and ARBs block the production and formation of angiotensin II, therefore improving kidney and cardiovascular diseases [5]. Reducing the intraglomerular pressure with an ACEI or ARB can minimize progression of, or even prevent, glomerular disease in the absence of glycemic control [6,7].

Several experimental, animal, and *in vivo* studies have shown that blockade of the RAAS with either ACEIs or ARBs leads to down-regulation of AGE, TGF-b, NADPH oxidase, ROS, reduced RAGE expression, reduced type IV collagen excretion, reduced mesangial extracellular matrix accumulation, reduced glomerulosclerosis, and albumin creatinine ratio [8,9,10].

These findings have been translated into several landmark clinical trials, demonstrating the beneficial effects of ACEIs and ARBs in DKD [8,11,12].

## 3. Single RAAS Blockade: Angiotensin-Converting Enzyme Inhibitor (ACEI) and Angiotensin II Receptor Blocker (ARB) Therapy

The rate of development of renal complications is thought to be more or less similar in type 1 (T1DM) and type 2 (T2DM) diabetes. However, after ten years of follow-up only 20% of T2DM patients with microalbuminuria progress to overt nephropathy in contrast to over 80% of T1DM patients. In addition, DKD can progress in the absence of albuminuria, suggesting that other tissue-destructive pathways might also have a role in the decline in renal function [13].

### 3.1. In Patients with Type 1 Diabetes (T1DM)

#### 3.1.1. ACEI Therapy

Since the beginning of their use, several studies have demonstrated that ACEI therapy promotes regression to normoalbuminuria, decreases progression to overt DKD, and slows the rate of progression in DKD [14,15], independently from their blood pressure-lowering effect [16]. In some patients ACEI have a marked antiproteinuric effect (with sustained long-term remission or regression of nephropathy and/or the nephrotic syndrome) and a good renal outcome [17,18,19,20]. This effects was seen in both hypertensive and normotensive subjects, and in patients with moderately-increased albuminuria [21,22], with overt nephropathy [8,23], and with advanced disease [24].

In 1993, the first trial to evaluate RAAS blockade on CKD progression was the *Collaborative Study Group trial* [8], performed in 409 T1DM patients with nephropathy (urine protein/creatinine ≥500 mg/g and baseline serum creatinine 1.5–2.5 mg/dL). Captopril (25 mg/8 h) strongly reduced the relative and absolute risks of the doubling of serum creatinine, whereas no significant benefit was observed among participants whose baseline serum creatinine was less than 1.5 mg/dL.

In 1994 *(European Microalbuminuria Captopril Study Group)* [21] and 1996 *(The Microalbuminuria Captopril Study Group)* [22], in two different trials performed in 317 patients with T1DM, moderately increased albuminuria, and a normal blood pressure; the patients were randomly assigned to captopril or placebo. Progression to overt proteinuria was markedly reduced after two years in the patients treated with captopril (7.6% *versus* 23.1%). In one of these trials [22], albumin excretion fell by 9.6% per year in patients receiving captopril compared to an increase of 14.2% per year with placebo.

In 1994, EUCLID *(EURODIAB Controlled Trial of Lisinopril in Insulin-Dependent Diabetes)* trial [25] was performed in 530 patients with T1DM and either moderately increased albuminuria (79 patients, mean albumin excretion rate 42 mcg/min) or normoalbuminuria (440 patients), randomly assigned to lisinopril (10 mg/d to 20 mg/d) or placebo. Among the patients with moderately increased albuminuria, the baseline albumin excretion fell with lisinopril and increased with placebo.

In 2005, a systematic review of 11 trials [26] of normotensive type 1 diabetic patients with moderately increased albuminuria, ACEI therapy significantly reduced the risk of progression to severely increased albuminuria (relative risk 0.36, 95% CI 0.22–0.58) and significantly increased the risk of regression to normoalbuminuria (relative risk 5.3, 95% CI 2.5–11.5).

#### 3.1.2. ARBs Therapy

Data are lacking on the efficacy of ARBs in patients with T1DM and moderately increased albuminuria. It seems likely that these drugs are as effective as ACEIs given their proven benefit in patients with T2DM and either moderately increased albuminuria or overt nephropathy.

There is no evidence that ACEIs or ARBs are effective for the primary prevention of moderately-increased albuminuria in T1DM patients who are normoalbuminuric and normotensive:

- In 2009, RASS *(Renin Angiotensin System Study)* [27] trial was performed in 285 normotensive normoalbuminuric T1DM patients, randomly assigned to receive losartan (100 mg/d) or enalapril (20 mg/d) or placebo and followed for five years. In addition, renal biopsy was performed at the study’s onset and after five years in 90% of the patients. Treatment with either losartan or enalapril had no effect compared to placebo on the fraction of glomerular volume occupied by the mesangium (the primary study end point) or other histologic findings seen in DKD. However, they found benefit on retinopathy progression of both enalapril and losartan as monotherapy over placebo.
- In 2009, DIRECT *(Diabetic Retinopathy Candesartan Trials)* [28] was performed in 3326 T1DM and 1905 T2DM normoalbuminuric patients, randomly assigned to candesartan (16 to 32 mg/d) or placebo. At a mean follow-up of 4.7 years, there was no difference between the candesartan and placebo groups in either the rate of developing moderately increased albuminuria (6% *versus* 5% and 16% *versus* 16% in the prevention and progression arms, respectively) or in the annual rate of increase in urinary albumin excretion. There was an overall trend to less severe retinopathy.

### 3.2. In Patients with Type 2 Diabetes (T2DM)

#### 3.2.1. In Normoalbuminuric T2DM Patients

Among T2DM patients with normal urine albumin excretion (<30 mg/24 h) and preserved GFR, RAAS blockade has been demonstrated to prevent the development of albuminuria in the presence of hypertension, although not without hypertension [27,29]. However, around 25% to 35% of diabetic patients with impaired GFR are normoalbuminuric [30].

##### 3.2.1.1. ACE Inhibitor Therapy

A similar benefit on the efficacy of ACEI therapy appears to be present in T2DM *versus* T1DM:

- In 1998, Ravid *et al.* [31] reported that daily treatment with enalapril compared with placebo reduced the risk of progression to microalbuminuria, decreased urinary albumin levels, and attenuated decline in renal function during 6 years of follow-up in 156 patients with T2DM.
- In the 2004, the BENEDICT *(Bergamo Nephrologic Diabetes Complication Trial)* study [29], was performed in 1204 normoalbuminuric T2DM patients, randomly assigned to trandolapril (2 mg/d) or placebo. At a follow-up of 3.6 years, trandolapril delayed the onset of microalbuminuria independently of blood pressure.
- In 2007, ADVANCE *(The Action in Diabetes and Vascular Disease: Preterax and Diamicron-MR Controlled Evaluation)* trial [32,33] was performed in 11,140 patients with T2DM and either normoalbuminura or microalbuminuria, randomly assigned to a fixed combination of perindopril-indapamide or placebo. After a mean of 4.3 years, the combination group present a significant reduction in blood pressure and in the risk of albuminuria progression (the rate of worsening or new onset of moderately increased albuminuria (19.6% *versus* 23.6%) and severely increased albuminuria). However, no clear effect of the therapy on decline in estimated glomerular filtration rate (eGFR) was observed.

##### 3.2.1.2. ARBs Therapy

In 2011, the ROADMAP *(Randomized Olmesartan and Diabetes Microalbuminuria Prevention)* trial [34] performed in 4447 patients with T2DM, showed that olmesartan was also effective in delaying the onset of microalbuminuria (8.2% *versus* 9.8%) independently of blood pressure. However, an unexplained excess of cardiovascular mortality was observed in the olmesartan group.

#### 3.2.2. In Microalbuminuric T2DM Patients

Among T2DM patients with microalbuminuria (30–300 mg/24 h), RAAS blockade has been demonstrated to reduce progression to macroalbuminuria (≥300 mg/24 h):

- In 2001, the IRMA-2 *(The Irbesartan in Patients with Type 2 Diabetes and Microalbuminuria Trial)* study [35] performed in 590 microalbuminuric T2DM patients, showed that treatment with irbesartan (150–300 mg/d) was associated with a dose-dependent reduction in risk of progression to macroalbuminuria, with an almost three-fold risk reduction with the highest dose (300 mg/d) at two years of follow-up. This effect was independent of the blood pressure-lowering properties of irbesartan.
- In 2007, in the INNOVATION *(Incipient to Overt: Angiotensin II Blocker, Telmisartan, Investigation on Type 2 Diabetic Nephropathy)* trial [36], telmisartan was associated with a lower transition rate to overt nephropathy than was placebo after one year of follow-up. In this trial, telmisartan also significantly reduced blood pressure levels. However, after adjustment for the difference in blood pressure levels between the placebo and treatment groups, the beneficial effect of telmisartan in delaying progression to overt nephropathy persisted.

#### 3.2.3. In Macroalbuminuric/Proteinuric T2DM Patients

Among T2DM patients with established DKD (macroalbuminuria and/or impaired GFR), RAAS blockade has been demonstrated to reduce risk of GFR loss, end-stage renal disease (ESRD), and death [8,11,12].

##### 3.2.3.1. ARBs Therapy

There is more data available on the preferential renoprotective efficacy of ARBs:

- In 2001, RENAAL *(The Reduction of Endpoints in NIDDM with the Angiotensin II Antagonist Losartan Study)* trial [12] was performed in 1513 T2DM patients with nephropathy (urine protein/creatinine ≥300 mg/g and a serum creatinine level of 1.3 to 3 mg/dL), randomly assigned to losartan (50–100 mg/d) or placebo. At 3.4 years, losartan reduced the incidence of a doubling of plasma creatinine by 25% and ESRD by 28%, and losartan reduced albuminuria by 28%, while the placebo was associated with a 4% increase in albuminuria, in the first six months. With *post hoc* analysis [37], albuminuria reduction by one-half was associated with a 36% decreased risk for the renal endpoint and 45% lower risk for the development of ESRD at follow-up and also showed that the incidence of ESRD was higher in Hispanic and Asian patients than in white and black patients and the losartan group has significant reductions in the development of and subsequent hospitalization for heart failure [38], neither study initially found significant cardiovascular mortality reduction. Benefits of losartan (with regard to prevention of the doubling of creatinine, ESRD, and death) were substantially greater among participants with higher level of baseline urine albumin/creatinine ratio (ACR). Greater baseline albuminuria, greater albuminuria six months after treatment, and lesser reduction in albuminuria from baseline to six months were each strongly associated with the primary composite end point of doubling of serum creatinine, ESRD, or death.
- In 2005, IDNT *(Irbesartan Diabetic Nephropathy Trial)* [11,39] study was performed in 1715 hypertensive T2DM patients with nephropathy (proteinuria ≥900 mg and a creatinine level of 1 to 3 mg/dL), randomly assigned to irbesartan (300 mg/d), amlodipine (10 mg/d), or placebo. At 2.6 years, irbesartan was associated with a risk of the primary endpoint (doubling of the plasma creatinine, development of ERSD, or death from any cause) that was 23% lower than amlodipine and 20% lower than placebo. A post-hoc analysis reported significantly lower levels of proteinuria were obtained with irbesartan (41% average decrease) than with amlodipine (11%) or control (16%) at one year. In addition, for the same proportional reduction in protein excretion, the risk reduction for renal failure was significantly greater for irbesartan compared with amlodipine. However the post hoc analysis of this trial showed that the risk of cardiovascular deaths for systolic blood pressure (SBP) less than 120 mmHg and DBP less than 85 mmHg was increased because of the high risk of all-cause mortality and myocardial infarction [40,41,42].
- In 2011, ORIENT *(Olmesartan Reducing Incidence of End-stage Renal Disease in Diabetic Nephropathy Trial)* trial [43], was performed in 577 Japanese and Chinese patients with T2DM and nephropathy, randomly assigned to olmesartan or placebo. At a mean follow-up of 3.2 years, olmesartan did not reduce the risk of the composite renal outcome (doubling of serum creatinine concentration, ESRD or death). However, 77% of the ORIENT study population was receiving ACEI therapy at baseline and this therapy was continued throughout the trial period. Thus, in the majority of participants, the effect of olmesartan added to ACEI therapy was tested rather than the effect of the ARB alone. This difference might explain the lack of renoprotection observed in this trial as opposed to in previous trials that used ACEI or ARB monotherapy. Moreover, an increased risk of cardiovascular events and hyperkalemia (9.2% *vs.* 5.3%) was observed in the olmesartan group.

##### 3.2.3.2. ACEI Therapy

ACEIs are at least as effective as ARBs in diabetic patients with established DKD.

- In 2004, DETAIL trial [44] was performed in 250 patients with nephropathy (82% moderately increased albuminuria and 18% severely increased albuminuria to a maximum of 1.4 g/d) and a baseline GFR of approximately 93 mL/min per 1.73 m^2^, randomly assigned to enalapril or telmisartan. At five years, there was a not significant decline in GFR in the enalapril group (14.9 *versus* 17.9 mL/min per 1.73 m^2^ with telmisartan). Both groups had similar rates or findings for the secondary endpoints (annual changes in the GFR, blood pressure, serum creatinine, urinary albumin excretion, end-stage kidney disease, cardiovascular events, and mortality).

### 3.3. In Patients with T1DM and T2DM

- In 2012, a meta-analysis by Hirst [45] of 49 randomized controlled trials involving 19,159 patients, analyzed the benefit of RAAS blockade according to the type of diabetes and the baseline albumin. In T1DM, RAAS blockade reduced urinary albumin excretion in patients with microalbuminuria, but not in those with normoalbuminuria. In T2DM, RAAS blockade reduced urinary albumin excretion in both groups of patients, with and without microalbuminuria.
- In 2012, another meta-analysis published by Nakao [46] of 19 randomized controlled trials (including *post hoc* analyses) involving 41,042 patients, reviewed whether RAAS blockade is beneficial for cardiovascular outcomes in patients with diabetes mellitus. RAAS blockade significantly reduced the risk of major cardiovascular events and myocardial infarction and there was no statistically significant trending towards fewer strokes and lower all-cause mortality.

Only a few long-term head-to-head studies have been designed to compare the effects of ARBs and ACEIs on the progression of DKD [44,47]. One such study with a follow-up of five years found that treatment with ARBs and ACEIs similarly decreased blood pressure and albuminuria and reduced the rate of GFR decline in 250 patients with T2DM and early-stage nephropathy [44].

In view of the existing evidence showing that RAAS blockade slows the progression of renal disease, ACEIs or ARBs are currently recommended for the treatment of early and late-stage DKD [48]. The beneficial effects of treatment with ACEIs and ARBs seem to be independent of the clinical stage of DKD. Whether early treatment with these medications is truly renoprotective (that is, associated with a significant reduction in risk of ESRD) still remains to be proven in a very large sample size or long follow-up prospective trial. However, patients with diabetes and advanced kidney disease are likely to progress relentlessly to ESRD despite treatment with ACEIs or ARBs, although more slowly.

## 4. Dual RAAS Blockade

### 4.1. Dual RAAS Blockade with ACEI and ARB

After multiple studies showed the kidney-protective effects of RAAS inhibitors in patients with DKD and overt proteinuria, a common hypothesis was that combination therapy with an ARB and an ACEI would result in more complete inhibition of RAAS and, therefore, offer greater kidney and cardiovascular protection. If RAAS blockade monotherapy is good, the logical next question is, would combination therapy by acting on different points in the RAAS system be better? [49].

The three pathophysiologic rationales for dual RAAS blockade are [50]:

1. Any single RAAS inhibitor does not completely block every step of the RAAS cascade. Greater down-regulation of the RAAS, as a whole, might be a consequence of RAAS blockade at different levels. In that order, potential lower doses of individual RAAS inhibitors could results in diminished adverse effects [50].
2. The “Aldosterone escape” was observed in many patients treated with a single RAAS blockade. Aldosterone baseline levels increased within 6–12 months in 30%–40% of the patients [51].
  - During chronic ACEI therapy, the increase of angiotensin I levels can be converted to angiotensin II, this leads to further generation of aldosterone: adding an ARB could counteract the effects of the residual angiotensin II.
  - During chronic ARB therapy, angiotensin II levels are elevated in addition to renin and angiotensin I, which can lead to angiotensin II competing with the ARB for the angiotensin II type 1 receptor, and angiotensin II binding to angiotensin II type 2 receptors on the adrenal glands, leading to the secretion of aldosterone [52].
  - Finally, plasma potassium increases due to both the ACEIs and the ARBs [50].
3. Many patients with macroalbuminuria despite being treated with single RAAS blockade presents “residual albuminuria”, setting a higher risk for the evolution of a chronic kidney disease, cardiovascular disease, and death [37,53]. Observations support the theory that reducing albuminuria will reduce the risk of long-term clinical outcomes, such as ESRD and death. Addition a second RAAS inhibitor may improve long-term outcome in DKD [50].

Nevertheless, is albuminuria causally related to progressive kidney and cardiovascular disease, or is it simply a marker of intrinsic risk that cannot be modified with therapy?

- There are other drugs that reduce albuminuria but do not improve long-term renal and cardiovascular outcomes.
- Diabetic nephropathy can progress in the absence of albuminuria, suggesting that other tissue-destructive pathways might also have a role in the decline in renal function [5].
- This uncertainty is reflected by the US FDA’s reluctance to accept albuminuria reduction as an end point for approving new therapies and leaves the optimal clinical response to residual albuminuria unclear [50].

In 1997, Menard *et al.* [54] first reported a synergistic effect between ACEIs and ARBs in spontaneously hypertensive rats, and suggested that these two drug classes would overcome the escape phenomenon resulting from incomplete blockade.

From 2000s, several trials with hard outcomes demonstrated dual RAAS blockade, compared with either therapy alone, improved control of blood pressure and may induce a greater reduction in protein excretion [55,56]. These effects have been appreciated in both type 1 and type 2 diabetes [55,57]:

- In 2000, Mogensen and colleagues [57] published the CALM *(Candesartan and Lisinopril Microalbuminuria)* study, performed in 199 hypertensive, T2DM and microalbuminuric patients. After 12 weeks of treatment, the combination of submaximal doses of lisinopril (20 mg), and candesartan (16 mg) led to a 50% reduction in albuminuria as compared with monotherapy in maximal doses, leading to a reduction of 24% and 39%, respectively. Dual therapy resulted in ~9–11 mmHg and 5–6 mmHg reduction in systolic and diastolic BPs, over monotherapy.
- In 2003, Jakobsen and colleagues [55], in 24 patients with T1DM and DKD, demonstrated a further 25% reduction in albuminuria when adding irbesartan (300 mg/d) during eight weeks compared with placebo on top of enalapril (40 mg/d). Four patients experienced transient hypotension during the dual blockade period.
- In 2007, IMPROVE *(The Irbesartan in the Management of Proteinuric Patients at High Risk for Vascular Events)* study [58], a randomized controlled trial in 405 patients with T2DM, hypertension, and albuminuria, failed to show a significant effect of combination therapy with ramipril and irbesartan (using maximal doses) on albuminuria as compared with monotherapy. However, there was no statistical difference in occurrence of adverse events, and hyperkalemia occurred in approximately 2.5% of patients in both groups.
- In 2008, Kunz and colleagues [59] published a meta-analysis of the effect of ACEI/ARB combination treatment on proteinuria: Over 1–4 months follow-up, analyzing seven clinical trials of ACEI plus ARB *versus* placebo plus ARB, the addition of an ACEI-reduced albuminuria by 24%; and analyzing seven clinical trials of ARB plus ACEI *versus* placebo plus ACEI, the addition of an ARB reduced albuminuria by 22%. The authors concluded that dual RAAS blockade seemed more effective in reducing proteinuria than either class of agent alone, but they also concluded that many of the smaller studies did not provide reliable data on adverse events, limiting the clinical applicability of the concept at that time.

In the case of dual RAAS blockade there was, for a long time, excitement about initial findings, and the treatment approach was adopted by many clinicians. Furthermore, as combination of ACEI and ARB has gained popularity in the primary care setting, the question of which patients now require withdrawal of combination therapy arises commonly [49]. It is important to realize that most of the initial positive results came from short-term studies using blood pressure and albuminuria reduction as outcome variables. Many of these initial studies were powered to establish the effect on these outcomes, but they may not have been powered to detect safety signals or may just not have had the proper duration to evaluate safety.

Despite the lack of robust evidence on long-term efficacy and safety, dual RAAS blockade has been embraced as a treatment option and has been recommended by experts and even in some clinical guidelines: 2007 ESH-ESC practice guidelines for the management of arterial hypertension: ESH-ESC Task Force on the management of arterial hypertension [60], ACCF/AHA Guidelines for the Diagnosis and Management of Heart Failure in Adults (2009), ESC guidelines for the diagnosis and treatment of acute and chronic heart failure (2012).

Enthusiasm regarding the promising effects of dual RAAS blockade on surrogate outcomes in small, short-term studies has been tempered by the disappointing results of large trials with hard outcomes. Randomized studies designed to test additional renal protection and safety, unexpectedly showed increased risk of adverse outcomes and events such as AKI, hyperkalemia, and/or need for dialysis:

- In 2008, the ONTARGET *(Ongoing Telmisartan Alone and in combination with Ramipril Global Endpoint)* trial [61,62] was performed in 25,620 patients older than 55 years with established cardiovascular disease (including 9603 patients with T2DM) and compare combination ramipril (10 mg/d) and telmisartan (80 mg/d) therapy with ramipril alone. In the subset of 3163 patients with DKD, combination therapy was associated with a similar death rate (2.3% *versus* 2.2%) and higher rates of acute kidney injury requiring dialysis (1.4% *versus* 0.8%), hyperkalemia (11.3% *versus* 7.8%), hypotension (2.8% *versus* 1.9%) and diarrhea. They conclude “…The combination of the two drugs was associated with more adverse events without an increase in benefit…”.
- In 2013, the VA NEPHRON-D *(Veterans Affairs Nephropathy in Diabetes)* trial [63], was performed in 1448 mostly male T2DM patients with DKD (macroalbuminuria and moderate-to-severe renal impairment (eGFR 30–90 mL/min/1.73m^2^) and compare combination losartan (100 mg/d) and lisinopril (10 to 40 mg/d) therapy with losartan alone. It must be discontinued after a median of 2.2 years because of a significantly higher frequency of acute kidney injury requiring hospitalization (18% *versus* 11%) or severe hyperkalemia (9.9% *versus* 4.4%). They conclude “…the results of our study show that the use of combination therapy with an ACE inhibitor and an ARB in patients with proteinuric diabetic kidney disease is associated with an increased risk of adverse events and does not provide an overall clinical benefit…”.

These data from large, independent clinical trials demonstrate that even in patients with established nephropathy, dual RAAS blockade fails to afford renoprotection. In addition, an interesting subgroup analysis of the ORIENT trial showed that olmesartan therapy provided renoprotection in the absence of background ACEI therapy [43]. The 16% relative reduction in renal risk with olmesartan reported in this analysis was similar to the effect size reported for losartan *versus* placebo in the RENAAL trial [12]. However, olmesartan therapy did not improve renal outcomes when given in combination with an ACEI (2% relative increase in renal risk) [64].

Thus, it seems that combination therapy with an ACEI plus an ARB should not be used in patients with DKD: it does not prevent renal disease progression or death, and it increases the rate of serious adverse events. Therefore, the authors of the *KDIGO clinical practice guidelines* discourage the use of dual RAAS blockade for the prevention and treatment of DKD [48].

### 4.2. Dual RAAS Blockade with ACEI/ARB and Direct Renin Inhibitor (DRI)

An increase in plasma renin activity during ACEI or ARB treatment as a result of compensatory mechanisms can limit the efficacy of these therapies [5]. As renin has an upstream role in the RAAS cascade, blockade of renin might inhibit the detrimental effects of both angiotensin II and aldosterone, resulting in additive beneficial hemodynamic and structural effects on the kidney. Based on this hypothesis, several renin inhibitors have been developed. Aliskiren does not reduce the production or secretion of renin from the juxtaglomerular apparatus, but directly blocks the activity of secreted renin and lowers plasma renin activity, the most proximal site of the RAAS cascade.

Initial exploratory studies in patients with DKD showed that aliskiren reduces proteinuria and blood pressure, independently of each other [65]. Moreover, the combination of aliskiren with an ARB led to a further reduction in albumin excretion compared to aliskiren or ARB alone.

- In 2008, AVOID *(Aliskiren in the Evaluation of Proteinuria in Diabetes)* trial [66], performed in 599 T2DM patients with hypertension and nephropathy (macroalbuminuria), randomly assigned to aliskiren (300 mg/d) or aliskiren plus losartan (100 mg/d). At 24 weeks of follow-up, combination therapy was associated with a significant 20% greater reduction in proteinuria and with a slight BP advantage but with no significant difference in the rate of decline in eGFR. Aliskiren therapy was associated with a significant increase in the risk of hyperkalemia.
- In 2012, ALTITUDE *(Aliskiren Trial in Type 2 Diabetes Using Cardiorenal Endpoints)* trial [67], performed in 8561 T2DM patients selected based on their high renal and cardiovascular risk profile, all receiving an ACEIs or ARBs at baseline, randomly assigned to aliskiren (300 mg/d) or placebo. After a median follow-up of 32.9 months, the incidence of kidney or cardiovascular events was similar with aliskiren and placebo (6% *versus* 5.9%) and adverse events requiring cessation of therapy (worsening of renal function, hyperkalemia, hypotension and diarrhea) were significantly more frequent (13.2% *versus* 10.2%) in the aliskiren group.
- In 2012, Harel and colleagues [68] published a meta-analysis of adverse events seen in studies of combination of aliskiren and other blockers of the RAAS. The authors analyzed data from 10 randomized controlled trials with 4814 patients and found a higher risk of hyperkalemia (relative risk 1.58, 95% confidence interval CI 1.24–2.02).
- In 2013, the VIVID study [69], performed in 1143 hypertensive patients with T2DM and Stage 1 to 2 CKD, using the combination of aliskiren/valsartan *vs.* valsartan monotherapy assessed blood pressure control. After two months combination therapy yielded significantly better blood pressure lowering. Safety events were similar in both groups, with no increased incidence of hyperkalemia in the combination therapy group.

Thus, the use of aliskiren in combination with either an ACEI or ARB does not appear to preserve renal function and increases the risk of adverse events, at least in advanced nephropathy. In April 2012, the FDA recommended a new contraindication against the use of aliskiren with ARBs or ACEIs in patients with diabetes because of the risk of renal impairment, hypotension, and hyperkalemia, and a warning to avoid the use of aliskiren with ARBs or ACEIs in patients with moderate to severe renal impairment (*i.e.*, where GFR <60 mL/min). The European Medicines Agency has issued similar warnings.

### 4.3. Dual RAAS Blockade with ACEI/ARB and Mineralocorticoid Receptor Antagonists (MRAs)

Several studies have shown that the addition of aldosterone receptor blockade to ACEI or ARB blockade can lead to further reduction in albuminuria.

Classically, aldosterone exerts its effects on volume status by the regulation of sodium reabsorption through the mineralocorticoid receptor (MR) on epithelial sodium channels, which are located on cortical collecting duct cells in the distal nephron. Additionally, there is increasing evidence that aldosterone is directly involved in the development and progression of renal disease via nonepithelial MR-mediated effects. Aldosterone exerts profibrotic effects through increased production of TGF-beta, reactive oxygen species, PAI-1 and increased collagen gene expression and synthesis, which can be abolished by MR blockade [70].

RAAS blockade initially decreases circulating aldosterone levels, but suppression will not be sustained in 10%–50% of patients, a phenomenon called “aldosterone breakthrough” [71]. This is particularly the case during long-term treatment or during sodium restriction, which potentiates the adrenal response to angiotensin II. Furthermore, “aldosterone breakthrough” is associated with a poor response to antiproteinuric treatment and an enhanced decline of renal function in patients with DKD [72]. It is suggested that the worst clinical prognosis of patients with “aldosterone breakthrough” is due to direct effects of aldosterone.

In line with this hypothesis, studies in patients with chronic kidney disease and early DKD show that MRA blockade on top of ACEI and/or ARB blockade exerts added renoprotective effects. Interestingly, the reduction in albuminuria induced by the addition of spironolactone to ACEI was related to aldosterone levels [72]. This suggests that aldosterone is a component of the renal damage that is associated with chronic kidney disease and that its inhibition by RAAS blockade can be incomplete.

In spite of these encouraging results on albuminuria, long-term data on the efficacy of MR blockade on hard endpoints, for example the development of ESRD or patient survival, are still lacking.

The eventual benefit of aldosterone blockade was then studied in various experimental models of diabetic nephropathy. MRAs appear to reduce proteinuria when used alone [73], and to have an additive effect on proteinuria when used in combination with an ACEI or an ARB in both type 1 and type 2 diabetes [72,74].

- In 2006, the only long-term study performed to date of add-on spironolactone therapy in diabetic and nondiabetic patients with proteinuria showed that the agent induced an initial acute fall in eGFR that predicted a later beneficial effect on decline in renal function and a remarkable and sustained reduction in proteinuria [75].
- In 2008, effects on albuminuria were summarized in a systematic review [71]. Fourteen studies evaluated spironolactone, whereas one study evaluated eplerenone. These MRAs were added to an ACEI in the majority of studies, although some studies added MRAs to an ARB or to an ACEI and an ARB (triple RAAS blockade). Compared with placebo or no additional intervention, most studies reported that addition of an MRA reduced albuminuria by 30%–40% (range, 15%–54%) but hyperkalemia is a concern particularly as renal function deteriorates. Currently treatment with this combination is not being recommended.
- In 2009, Medhi and colleagues published a placebo-controlled trial [74] performed in 81 patients with T1DM or T2DM, hypertension, and albuminuria ≥300 mg/g, all receiving high doses of lisinopril (80 mg/d) at baseline, randomly assigned to spironolactone (25 mg/d), losartan (100 mg/d), or placebo. At 48 weeks of follow-up, patients treated with spironolactone had a 34% decrease in urine albumin-to-creatinine ratio compared to placebo while patients who received losartan had only a 17% decrease not significantly different from placebo, despite a similar effect on blood pressure and serum potassium levels. A direct statistical comparison of spironolactone *versus* losartan was not reported.
- In 2006, Epstein and colleagues examined the efficacy and safety of eplerenone in a trial [76] of 268 patients with T2DM and CKD stage 1–2 (eGFR 74 mL/min per 1.73 m^2^), already treated with an ACEI randomly assigned to eplerenone (50 or 100 mg/d) or placebo. Patients treated with eplerenone therapy present a significant reduction in albuminuria (40%–50% *versus* <10%). Severe hyperkalemia (>6 meq/L) occurred in 9% and 23% of patients receiving 50 and 100 mg/d of eplerenone, respectively, compared with 12% of patients in the placebo group.
- In 2014, a meta-analysis of eight trials and 404 patients [77], combined treatment ACEI/ARB and MRA further reduced albuminuria by 23 to 61%, increased hyperkalemia prevalence and slightly decreased eGFR values, compared with standard treatment.

There is no long-term data regarding the benefit with the combination of ACEI or ARB and MRA in terms of slowing the rate of loss of GFR, so the role of the aldosterone blocker is unclear in CKD progression. Reduction in albuminuria is not a validated surrogate for slowing nephropathy progression and the risk of inducing or aggravating hyperkalemia may limit the use of aldosterone antagonists although the risk of hyperkalemia may possibly be lower with an ARB [78]. Whether the benefits of combined treatment by an ACEI/ARB and an MRA exceed the risks of this association has to be confirmed by an adequately-powered prospective, comparative, clinical trial.

With the publication of recent endpoint trials, the tide has shifted because of higher rates of side effects and no clear benefit. Although it can be argued that the ONTARGET study population is heterogeneous with a cardiovascular rather than kidney risk profile, that the ALTITUDE study is only investigating one possible combination of RAAS treatment, and that the VA NEPHRON D study was much smaller, the conclusion so far on the basis of three hard endpoint studies must be, until hard endpoint trials tells us otherwise or until we know more about which patients to select for combination treatment, that any combination using renin inhibition, ACEIs, or ARBs is not advisable. In the case of combinations with aldosterone receptor blockade, hard endpoint trials are still lacking; however, more data and information on this topic are sure to come [79].

## 5. Combination ACEI/ARB and Calcium Channel Blockers

Nondihydropyridine calcium channel blockers (diltiazem and verapamil) appear to be as effective as an ACEI or ARB in lowering protein excretion in diabetic patients [32,80,81,82]. Furthermore, the antiproteinuric effects of verapamil and an ACEI may be additive.

- In a study [80] of 30 patients with T2DM in which lisinopril (mean dose 29 mg/d) or verapamil (mean dose 360 mg/d) alone lowered protein excretion from 5.8 to 2.7 g/d. Low-dose combination of both drugs (mean of 16 mg of lisinopril and 187 mg of verapamil) had a much greater antiproteinuric effect (down from 6.8 to 1.7 g/d) but was also associated with fewer drug-induced side effects (such as constipation with verapamil and dizziness with lisinopril).
- A similar antiproteinuric effect has been demonstrated with combination therapy with verapamil and trandolapril [83]. But their potential efficacy in the preservation of renal function in relation to ACEIs has not yet been evaluated in humans.

Dihydropyridine calcium channel blockers (amlodipine, nifedipine, nitrendipine) have a variable effect ranging from increased protein excretion to no effect to a fall in protein excretion in different studies [11,39,80,81].

## 6. Combination ACE Inhibitor/ARB and Salt Restriction

RAAS blockade is largely ineffective during states of volume excess or increased sodium intake. Sodium restriction and diuretic treatment increase the top of the dose-response curve to RAAS blockade, and therefore a larger maximum response can be obtained [84].

- High sodium intake has been found to blunt the antiproteinuric as well as the antihypertensive response to ACE inhibition, in albuminuric patients.
- Moderate salt restriction to 5–6 g/d (recommended by KDIGO 2012 Clinical Practice Guideline for the Evaluation and Management of CKD [48]) has been found to enhance the antihypertensive and antiproteinuric effects of RAAS blockade [85]. Their addition to ACEI was significantly more effective than dual RAAS blockade [86], as effective as the addition of diuretics, even in patients resistant to RAAS blockade [84]. Into the bargain, low-sodium diet improves the long-term cardioprotective and renal efficacy of single RAAS blockade [87]. In a *post hoc* analysis of the RENAAL and IDNT trials [87], ARB provided the best renoprotective effects in patients who had the lowest tertile of sodium intake: the risk of renal events was reduced by 43% or increased by 37% in patients with the lowest and highest tertile of sodium intake, respectively.
- Extremely aggressive sodium restriction on top of single or dual RAAS blockade might elicit adverse renal and cardiovascular events. A meta-analysis from Italy [88] concluded *increased* mortality in patients with heart failure on a very strict sodium diet treated with daily diuretics and fluid restriction, but this paper was retracted soon after publication because of concern over reporting of duplicate data and the inability of the authors to provide the original data for verification.
- If a low-sodium diet is not possible, diuretic therapy with increased furosemide dosage partially corrects the loss of antiproteinuric effect due to a high sodium intake, even in nephrotic patients [89].

## 7. What is and What Should be the Current Use of RAAS Blockade?

U.S. Renal Data System (USRDS) 2013 Annual Data reported that anti-RAAS therapy, the single most important therapy for DKD, may be underutilized [90]. Despite the beneficial effects shown in many long-term randomized, controlled, trials, some clinicians are reluctant to start RAAS blockade in patients with diabetic nephropathy.

The usage of anti-RAAS treatment decreases as CKD advances. There are few reasons not to maximize anti-RAAS therapy, especially given the fact that these agents are beneficial in reducing albuminuria, in delaying the progression of DKD, in control hypertension, and in heart failure (a common companion of hypertensive DKD patients) [91]. Paradoxically, the population that would benefit most from anti-RAAS therapy is the one denied the treatment [92].

The main reasons wielded by some clinicians not to use RAAS blockade in DKD patients are Table 2 [92]:

**Table 2:** Main reasons wielded by some clinicians not to use RAAS blockade in DKD patients.

Reasons Wielded by Some Clinicians Not to Use RAAS Blockade in DKD Patients	Arguments against Reasons Wielded by some Clinicians for Not Using RAAS Blockade in DKD Patients
*“Anti-RAAS therapy has no advantage to other therapies”*	■ The RAAS is currently the primary therapeutic target for the prevention and treatment of DKD. High-quality animal experimental and human studies clearly demonstrate that RAAS overactivity plays a central role in the pathogenesis of DKD. Anti-RAAS therapy is potentially salutary by reducing albuminuria and, thereby, interstitial inflammation, the most important predictor of a kidney’s longevity [92]. ■ Blockade of the RAAS is a proven cornerstone of therapy for the prevention and treatment of DKD. An elegant body of scientific accomplishment from basic science through clinical trials has solidified the use of ACEIs, ARBs, and other RAAS inhibitors in patients with diabetes [51].
*“Anti-RAAS therapy can accelerate the progression rate of kidney decline”* [92]	■ Since the advent of ACEI therapy it has been acknowledged that a decline in GFR may occur. The initiation of RAAS blockade is often followed by an acute fall in GFR [93,94]. Although this acute decline in renal function might be a concern, drug withdrawal or dose lowering should only be considered if hyperkalemia develops or GFR continues to decline. Importantly, studies with long-term follow-up have shown that the initial fall in GFR in response to initiation of RAAS blockade is inversely correlated with the long-term decline in renal function; patients whose GFR declined shortly after starting RAAS blockade had a slower rate of decline in renal function during follow-up than did those whose GFR did not initially decrease [94,95]. Some degree of serum creatinine elevation must be anticipated and tolerated. In fact, if the DKD patient’s serum creatinine does not escalate after institution of anti-RAAS treatment, suspect that something is amiss. ■ Clinical circumstances, under which kidney injury can develop during RAAS blockade, include hypotension, dehydration, clinically inapparent volume overload, high salt ingestión, nonsteroidal anti-inflammatory drug (NSAID) intake, renal artery stenosis, exposure to contrast agents, and hospitalization. Onuigbo has described this phenomenon in great detail [96,97,98,99]. These states of kidney injury might exacerbate CKD resulting in ESRD.
*“Intensive blood pressure-lowering is dangerous in diabetic patients”* [92]	■ From 1990s it became clear that RAAS blockade confers additional renoprotection beyond what could be expected from blood pressure lowering alone [8,11,100]. However, not all trials have demonstrated this point: In the UKPDS trial [101], captopril therapy decreased blood pressure levels to the same extent as did the β-blocker atenolol but did not provide additional renoprotection. The ABCD *(The Appropriate Blood Pressure Control in Diabetes)* trial fail to show a benefit on further slowing of CKD progression at a blood pressure of less than 130/80 mmHg even with a RAAS blocker. From data from the ACCORD *(Action to Control Cardiovascular Risk in Diabetes)* trial [102], practitioners have likely relaxed their ambitions to tightly controlling diabetic patients’ blood pressures. ■ However, although intensive blood pressure-lowering is not efficacious in diabetes (140 *vs.* 120 mmHg) for serious cardiovascular events [103], the continued application of anti-RAAS therapy may still be advantageous. ■ Since ACEI inhibition is approved as a measure for secondary stroke prevention, ongoing anti-RAAS blood pressure-lowering may be protective for stroke, but the blood pressure need only be reduced to 140 mmHg.
*“Anti-RAAS therapy is dangerous in elderly patients”*	■ The recently released JNC 8 guidelines advise caution in the use of anti-RAAS therapy in the elderly due to the risk of increasing the serum creatinine and hyperkalemia. Stringent adherence to this guideline may leave those with proteinuria and those who could tolerate anti-RAAS therapy at risk [92,104].
*“Anti-RAAS therapy can induce potential lethal hyperkalemia”* [92]	■ Avoidance of anti-RAAS therapy to avoid hyperkalemia is not well supported by literature. It exists, but it is far less common than believed. In addition, hyperkalemia at the level of 5.0–5.5 meq/L is not dangerous: Elevations of the baseline serum potassium from per se 4.5 meq/L by 0.5 and 1.0 meq/L only increase the membrane threshold potential by 3% and 6%, respectively, clinically irrelevant in the absence of an extremely low serum ionized calcium concentration [92]. ■ Single RAAS Blockade generally will not lead to hyperkalemia: The frequency of hyperkalemia was only 1.6% at GFR levels greater than 40 mL/min per 1.73 m^2^. In the absence anti-RAAS therapy, hiperkalemia do not occur until the GFR is nearly 15 mL/min per 1.73 m^2^. When hyperkalemia occurs in a diabetic individual, the diagnosis of type 4 renal tubular acidosis or hyperkalemic, hyperchloremic acidosis from hyporeninemic hypoaldosteronism is frequently invoked, but it can be excluded another causes (subclinical volume depletion, undisclosed obstructive uropathy, decreased effective circulatory volume from decompensated heart failure, drugs…). ■ With dual RAAS Blockade (ACEI plus ARB or ACEI plus spironolactone) hyperkalemia does occur more frequently [74]. Since the risk of hyperkalemia has been the ‘‘Achilles heel’’ of all combination studies, three separate studies have evaluated predictors of hyperkalemia, and all share similar findings: Those patients with an eGFR <45 mL/min per 1.73 m^2^ who have a baseline potassium ≥4.5 mEq/L while already on an appropriate diuretic and a RAS blocker should not be given a second RAAS blocker because of a more than three-fold risk of developing hyperkalemia [105,106].
*“Anti-RAAS therapy not prevent residual risk of ESRD”*	Although RAAS therapy slows the progression of DKD to ESRD, the residual risk of ESRD remains high and correlates with the residual level of albuminuria in patients receiving this therapy [107]. It could be the result of: ■ Insufficient RAAS blockade attributable to insufficient dosing or compensatory feedback responses [5]: As ACEIs and ARBs are registered as antihypertensive drugs, the doses that are frequently used in clinical practice are the maximum recommended for blood-pressure reduction. However, studies have shown that use of “supra-maximal doses” of this drugs result in further lowering of albuminuria, in the presence or absence of additional changes in blood pressure: ○ In ROAD *(Renoprotection of Optimal Antiproteinuric Doses)* study [108] performed in 360 non-diabetic Chinese patients with proteinuria and chronic renal insufficiency, the optimal antialbuminuric and tolerable doses of benazepril or losartan (compared with the conventional doses) resulted in an additional reduction in albuminuria and a 50% reduction in the risk of ESRD during 3.7 years of follow-up. ○ In 52 patients [109] with T2DM and microalbuminuria, treatment with supra-maximal doses of irbesartan (up to 900 mg/d) resulted in a 15% greater reduction in albuminuria at two months than did treatment with the maximum recommended blood pressure lowering dose (300 mg/d). ○ In 269 patients [110] with baseline proteinuria ≥1 g/d (54% of whom had diabetes), compared with a standard antihypertensive candesartan dose of 16 mg/d, doses of 64 and 128 mg/d (each above the accepted maximal antihypertensive dose) reduced urine protein excretion by 16% and 30% after 30 weeks, respectively. ■ To the involvement of pathways that are not affected by RAAS blockade, such as endothelin pathways and specific inflammatory profibrotic pathways.

## 8. Conclusions

According to previous studies and the current KDIGO recommendations [5,48,92,111,112,113] we can conclude:

- All patients with DKD should be started on RAAS blockers.
- It is essential to individualize blood pressure (BP) targets and treatment regimens according to age, coexistent cardiovascular disease, and other comorbidities, risk of progression of CKD, presence or absence of retinopathy, other therapies and tolerance of treatment, with gradual escalation of treatment and close attention to adverse events related to BP treatment, including electrolyte disorders, acute deterioration in kidney function, orthostatic hypotension, and drug side effects.
- There is insufficient evidence to recommend combining an ACE-I with ARBs to prevent progression of CKD.
- Patients with low or moderate risk for progressive kidney disease (normo/microalbuminuria with normal estimated GFR):
  ○ In normotensive normoalbuminuric T1DM and T2DM patients, RAAS-inhibiting agents for primary prevention is not justified.
  ○ In normotensive microalbuminuric T1DM and T2DM patients, an ARB or ACEI must be used.
  ○ In hypertensive microalbuminuric T1DM and T2DM patients, ACEI or ARB monotherapy remains the mainstay of treatment.
  ○ Due to the lack of evidence supporting a target blood pressure of less than 130/80 mmHg, we recommend a goal of 140/90 mmHg.
  ○ The use of RAAS blockers and a blood pressure of less than 130/80 mmHg is not of proven value for slowing CKD progression.
  ○ Dual RAAS blockade should not be routinely applied.
- Patients at high risk (macroalbuminuria or impaired GFR):
  ○ All patients should be treated on RAAS blockers especially those with advanced Stage 3 or higher CKD.
  ○ Hypertensive T1DM and T2DM patients must be treated with BP-lowering drugs to maintain a lower blood pressure of less than 130/80 mmHg especially those with more than 1 g of proteinuria and advanced Stage 3 or higher CKD.
  ○ Maintaining anti-RAAS therapy for its secondary stroke prevention and antiproteinuric effects must always remain a consideration.
  ○ It is recommended that temporary discontinuation of RAAS blockers in patients with GFR <60 mL/min/1.73 m2 who have serious intercurrent illness (particularly in a setting of dehydration such as diarrhoea and vomiting) that increases the risk of acute kidney failure.
  ○ Clinicians should carefully weigh the potential benefits of dual RAAS blockade against potential adverse effects including hyperkalemia on an individual basis.

There are now well-powered primary and secondary trials clearly showing that combined RAAS blockade does not alter the natural history of DKD. Although RAAS blockade has formed the cornerstone of pharmacological management in diabetic nephropathy over the last two decades, we have continued to witness progression of CKD to ESRD. This is not surprising given that they do not block the multiple pathophysiological pathways leading to DKD. It is therefore necessary to look beyond this single pathway in strategies to prevent what is a complex and multifactorial condition.

However, “the devil is in the detail” as says Wong [49] in a revision of the current “state of play” for dual RAAS blockade: There are a number of caveats and clinical scenarios where the conclusion may not be valid and to assume so may be to the detriment of the patient [49]:

- Very hypertensive patient (>160/100 mmHg), excluded from ONTARGET trial, requiring multi-agent therapy (including ACEI/ARB in combination), where CVD risk is directly correlated with BP, any BP lowering would be expected to translate to a significant reduction in CVD-related morbidity and mortality. In a case control study [114] of 600 patients, it has been found that there is no excess decline in renal function in the very hypertensive patient (>160/100 mmHg) treated with combination of ACEI and ARB in comparison to monotherapy. This suggests the primacy of BP control in preserving renal function over any potential decline afforded by combination therapy in this very hypertensive at-risk group.
- Some of the ONTARGET patients were using therapies proven to have beneficial effects on vascular risk (such as β-blockers, statin, and anti-platelet treatment), attenuating any additional benefit from combination ACEI/ARB therapy. However, in clinical practice, many patients do not or cannot take statin treatment and cannot use β-blockers or anti-platelet agents. It is theoretically possible that a greater RAAS blockade would be beneficial in this subgroup of patients who cannot have risk attenuated by other pharmacological means.

“It would still be premature to sound the death knell for dual blockade” [49]. Lifestyle interventions or diagnostic tools might be used to optimize RAAS blockade and identify those patients who are most likely to benefit from the therapy [5]. The modern catchcry of clinical medicine is individualization of therapy, but we are hamstrung by the inability to measure completeness of RAAS blockade in an individual.

Hence, there remains a clear and growing need for emerging therapeutic strategies and, indeed, there are several agents currently under investigation, targeting other metabolic pathways.

Several recent candidates have failed to meet expectations or are now being tested for therapy of DKD: prorenin receptor antagonists, inhibitors of AGE formation (aminoguanidine, pimagedine, pyridoxamine), aldose reductase inhibitors (sorbinol and tolerestat), triterpenoid activator of the transcription factor Nrf2 (bardoxolone methyl), glycosaminoglycan (sulodexide), selective vitamin D receptor activator (Paricalcitol), ET receptor blockers (avosentan, atrasentan, sitaxsentan), antioxidant therapy (vitamin A, vitamin C, vitamin E, N-acetyl cysteine, b-carotenes, flavonoids…), PKC-b inhibitor (ruboxistaurin), inhibitors of TGF-b production (pirfenidone), recombinant human anti-CTGF monoclonal immunoglobulin G antibody (FG-3019), methyl xanthine derivative (pentoxifylline), anti-TNF-a antibody (Infliximab), inhibitors of Janus kinases (JAK), AGE-crosslink breaker (alagebrium), non-steroidal mineralocorticoid receptor antagonist (PF-03882845, BAY 94-8862).

The optimal qualities required from an effective novel agent in DKD would be clear additive effects to the benefits of renin-angiotensin aldosterone blockade, tolerability, safety and, ideally, benefits in terms of cardiovascular outcomes.

## References

[1] Williams J.S., Williams G.H. (2003). 50th anniversary of aldosterone. J. Clin. Endocrinol. Metab..

[2] Ferreira S.H. (1965). A bradykinin-potentiating factor (BPF) present in the venom of bothrops jararaca. Br. J. Pharmacol. Chemother..

[3] Ng K.K., Vane J.R. (1970). Some Properties of Angiotensin Converting Enzyme in the Lung *in vivo*. Nature.

[4] Gradman A., Schmieder R., Lins R., Nussberger J., Chiang Y., Bedigian M. (2005). Aliskiren, a novel orally effective renin inhibitor, provides dose-dependent antihypertensive efficacy and placebo-like tolerability in hypertensive patients. Circulation.

[5] Roscioni S.S., Heerspink H.J., Zeeuw D. (2014). The effect of RAAS blockade on the progression of diabetic nephropathy. Nat. Rev. Nephrol..

[6] Anderson S., Rennke H.G., Garcia D.L., Brenner B.M. (1989). Short and long term effects of antihypertensive therapy in the diabetic rat. Kidney Int..

[7] Fliser D., Wagner K.K., Loos A., Tsikas D., Haller H. (2005). Chronic angiotensin II receptor blockade reduces (intra)renal vascular resistance in patients with type 2 diabetes. J. Am. Soc. Nephrol..

[8] Lewis E.J., Hunsicker L.G., Bain R.P., Rohde R.D. (1993). The effect of angiotensin-converting-enzyme inhibition on diabetic nephropathy. N. Engl. J. Med..

[9] Furukawa M., Gohda T., Tanimoto M., Tomino Y. (2013). Pathogenesis and novel treatment from the mouse model of type 2 diabetic nephropathy. Sci. World J..

[10] Andersen S., Tarnow L., Rossing P., Hansen B.V., Parving H.H. (2000). Renoprotective effects of angiotensin II receptor blockadein type 1 diabetic patients with diabetic nephropathy. Kidney Int..

[11] Lewis E.J., Hunsicker L.G., Clarke W.R., Berl T., Pohl M.A., Lewis J.B., Ritz E., Atkins R.C., Rohde R., Raz I., Collaborative Study Group (2001). Renoprotective effect of the angiotensin-receptor antagonist irbesartan in patients with nephropathy due to type 2 diabetes. N. Engl. J. Med..

[12] Brenner B.M., Cooper M.E., de Zeeuw D., Keane W.F., Mitch W.E., Parving H.H., Remuzzi G., Snapinn S.M., Zhang Z., Shahinfar S. (2001). Effects of losartan on renal and cardiovascular outcomes in patients with type 2 diabetes and nephropathy. N. Engl. J. Med..

[13] Perkins B.A., Ficociello L.H., Roshan B., Warram J.H., Krolewski A.S. (2010). In patients with type 1 diabetes and new-onset microalbuminuria the development of advanced chronic kidney disease may not require progression to proteinuria. Kidney Int..

[14] Kasiske B.L., Kalil R.S., Ma J.Z., Liao M., Keane W.F. (1993). Effect of antihypertensive therapy on the kidney in patients with diabetes: A meta-regression analysis. Ann. Intern. Med..

[15] Parving H.H., Hommel E., Jensen B.R., Hansen H.P. (2001). Long-term beneficial effect of ACE inhibition on diabetic nephropathy in normotensive type 1 diabetic patients. Kidney Int..

[16] Hoogwerf B.J., Young J.B. (2000). The HOPE study. Ramipril lowered cardiovascular risk, but vitamin E did not. Clevel. Clin. J. Med..

[17] Wilmer W.A., Hebert L.A., Lewis E.J., Rohde R.D., Whittier F., Cattran C., Levey A.S., Lewis J.B. (1999). Remission of nephrotic syndrome in type 1 diabetes: Long-term follow-up of patients in the Captopril Study. Am. J. Kidney Dis..

[18] Hovind P., Rossing P., Tarnow L., Smidt U.M., Parving H.H. (2001). Remission and regression in the nephropathy of type 1 diabetes when blood pressure is controlled aggressively. Kidney Int..

[19] Hovind P., Rossing P., Tarnow L., Smidt U.M., Parving H. (2001). Remission of nephrotic-range albuminuria in type 1 diabetic patients. Diabetes Care.

[20] Hovind P., Tarnow L., Rossing P., Carstensen B., Parving H.H. (2004). Improved survival in patients obtaining remission of nephrotic range albuminuria in diabetic nephropathy. Kidney Int..

[21] Viberti G., Mogensen C.E., Groop L.C., Pauls J.F. (1994). Effect of captopril on progression to clinical proteinuria in patients with insulin-dependent diabetes mellitus and microalbuminuria. JAMA.

[22] (1996). Captopril reduces the risk of nephropathy in IDDM patients with microalbuminuria. Diabetologia.

[23] Hebert L.A., Bain R.P., Verme D., Cattran D., Whittier F.C., Tolchin N., Rohde R.D., Lewis E.J., the Collaborative Study Group (1994). Remission of nephrotic range proteinuria in type I diabetes. Kidney Int..

[24] Steffes M.W. (2001). Affecting the decline of renal function in diabetes mellitus. Kidney Int..

[25] Chaturvedi N., the EUCLID Study Group (1997). Randomised placebo-controlled trial of lisinopril in normotensive patients with insulin-dependent diabetes and normoalbuminuria or microalbuminuria. Lancet.

[26] Newman D.J., Mattock M.B., Dawnay A.B., Kerry S., McGuire A., Yaqoob M., Hitman G.A., Hawke C. (2005). Systematic review on urine albumin testing for early detection of diabetic complications. Health Technol. Assess..

[27] Mauer M., Zinman B., Gardiner R., Suissa S., Sinaiko A., Strand T., Drummond K., Donnelly S., Goodyer P., Gubler M.C. (2009). Renal and retinal effects of enalapril and losartan in type 1 diabetes. N. Engl. J. Med..

[28] Bilous R., Chaturvedi N., Sjølie A.K., Fuller J., Klein R., Orchard T., Porta M., Parving H.H. (2009). Effect of candesartan on microalbuminuria and albumin excretion rate in diabetes: Three randomized trials. Ann. Intern. Med..

[29] Ruggenenti P., Fassi A., Ilieva A.P., Bruno S., Iliev I.P., Brusegan V., Rubis N., Gherardi G., Arnoldi F., Ganeva M. (2004). Preventing microalbuminuria in type 2 diabetes. N. Engl. J. Med..

[30] Kramer H.J., Nguyen Q.D., Curhan G., Hsu C.Y. (2003). Renal insufficiency in the absence of albuminuria and retinopathy among adults with type 2 diabetes mellitus. JAMA.

[31] Ravid M., Brosh D., Levi Z., Bar-Dayan Y., Ravid D., Rachmani R. (1998). Use of enalapril to attenuate decline in renal function in normotensive, normoalbuminuric patients with type 2 diabetes mellitus. A randomized, controlled trial. Ann. Intern. Med..

[32] Patel A., MacMahon S., Chalmers J., Neal B., Woodward M., Billot L., Harrap S., Poulter N., Marre M., ADVANCE Collaborative Group (2007). Effects of a fixed combination of perindopril and indapamide on macrovascular and microvascular outcomes in patients with type 2 diabetes mellitus (the ADVANCE trial): A randomised controlled trial. Lancet.

[33] De Galan B.E., Perkovic V., Ninomiya T., Pillai A., Patel A., Cass A., Neal B., Poulter N., Harrap S., Mogensen C.E. (2009). Lowering blood pressure reduces renal events in type 2 diabetes. J. Am. Soc. Nephrol..

[34] Haller H., Ito S., Izzo J.L., Januszewicz A., Katayama S., Menne J., Mimran A., Rabelink T.J., Ritz E., Ruilope L.M. (2011). ROADMAP Trial Investigators Olmesartan for the delay or prevention of microalbuminuria in type 2 diabetes. N. Engl. J. Med..

[35] Parving H.H., Lehnert H., Bröchner-Mortensen J., Gomis R., Andersen S., Arner P. (2001). The effect of irbesartan on the development of diabetic nephropathy in patients with type 2 diabetes. N. Engl. J. Med..

[36] Makino H., Haneda M., Babazono T., Moriya T., Ito S., Iwamoto Y., Kawamori R., Takeuchi M., Katayama S., INNOVATION Study Group (2007). Prevention of transition from incipient to overt nephropathy with telmisartan in patients with type 2 diabetes. Diabetes Care.

[37] De Zeeuw D., Remuzzi G., Parving H.H., Keane W.F., Zhang Z., Shahinfar S., Snapinn S., Cooper M.E., Mitch W.E., Brenner B.M. (2004). Proteinuria, a target for renoprotection in patients with type 2 diabetic nephropathy: Lessons from RENAAL. Kidney Int..

[38] Remuzzi G., Ruggenenti P., Perna A., Dimitrov B.D., de Zeeuw D., Hille D.A., Shahinfar S., Carides G.W., Brenner B.M., RENAAL Study Group (2004). Continuum of renoprotection with losartan at all stages of type 2 diabetic nephropathy: A *post hoc* analysis of the RENAAL trial results. J. Am. Soc. Nephrol..

[39] Atkins R.C., Briganti E.M., Lewis J.B., Hunsicker L.G., Braden G., Champion de Crespigny P.J., DeFerrari G., Drury P., Locatelli F., Wiegmann T.B. (2005). Proteinuria reduction and progression to renal failure in patients with type 2 diabetes mellitus and overt nephropathy. Am. J. Kidney Dis..

[40] Berl T., Hunsicker L.G., Lewis J.B., Pfeffer M.A., Porush J.G., Rouleau J.L., Drury P.L., Esmatjes E., Hricik D., Pohl M. (2005). Impact of achieved blood pressure on cardiovascular outcomes in the Irbesartan Diabetic Nephropathy Trial. J. Am. Soc. Nephrol..

[41] Pohl M.A., Blumenthal S., Cordonnier D.J., De Alvaro F., Deferrari G., Eisner G., Esmatjes E., Gilbert R.E., Hunsicker L.G., de Faria J.B. (2005). Independent and additive impact of blood pressure control and angiotensin II receptor blockade on renal outcomes in the irbesartan diabetic nephropathy trial: Clinical implications and limitations. J. Am. Soc. Nephrol..

[42] Berl T., Hunsicker L.G., Berl T., Hunsicker L.G., Lewis J.B., Pfeffer M.A., Porush J.G., Rouleau J.L., Drury P.L., Esmatjes E., Hricik D., Parikh C.R. (2003). Cardiovascular outcomes in the Irbesartan Diabetic Nephropathy Trial of patients with type 2 diabetes and overt nephropathy. Ann. Intern. Med..

[43] Imai E., Chan J.C., Ito S., Yamasaki T., Kobayashi F., Haneda M., Makino H. (2011). Effects of olmesartan on renal and cardiovascular outcomes in type 2 diabetes with overt nephropathy: A multicentre, randomised, placebo-controlled study. Diabetologia.

[44] Barnett A.H., Bain S.C., Bouter P., Karlberg B., Madsbad S., Jervell J., Mustonen J., Diabetics Exposed to Telmisartan and Enalapril Study Group (2004). Angiotensin-receptor blockade *versus* converting-enzyme inhibition in type 2 diabetes and nephropathy. N. Engl. J. Med..

[45] Hirst J.A., Taylor K.S., Stevens R.J., Blacklock C.L., Roberts N.W., Pugh C.W., Farmer A.J. (2012). The impact of renin-angiotensin-aldosterone system inhibitors on Type 1 and Type 2 diabetic patients with and without early diabetic nephropathy. Kidney Int..

[46] Nakao Y.M., Teramukai S., Tanaka S., Yasuno S., Fujimoto A., Kasahara M., Ueshima K., Nakao K., Hinotsu S., Nakao K. (2012). Effects of renin-angiotensin system blockades on cardiovascular outcomes in patients with diabetes mellitus: A systematic review and meta-analysis. Diabetes Res. Clin. Pract..

[47] Lacourciere Y., Bélanger A., Godin C., Hallé J.P., Ross S., Wright N., Jean M.J. (2000). Long-term comparison of losartan and enalapril on kidney function in hypertensive type 2 diabetics with early nephropathy. Kidney Int..

[48] (2013). Kidney Disease: Improving Global Outcomes (KDIGO) CKD Work Group. KDIGO 2012. Clinical Practice Guideline for the Evaluation and Management of Chronic Kidney Disease. Kidney Int. Suppl..

[49] Wong J. (2013). Is there benefit in dual renin-angiotensin-aldosterone system blockade? No, yes and maybe: A guide for the perplexed. Diabates Vasc. Dis. Res..

[50] Pichler R.H., de Boer I.H. (2010). Dual renin-angiotensin-aldosterone system blockade for diabetic kidney disease. Curr. Diabetes Rep..

[51] Bomback A.S., Klemmer P.J. (2007). The incidence and implications of aldosterone breakthrough. Nat. Clin. Pract. Nephrol..

[52] Forclaz A., Maillard M., Nussberger J., Brunner H.R., Burnier M. (2003). Angiotensin II receptor blockade: Is there truly a benefit of adding an ACE inhibitor?. Hypertension.

[53] De Boer I.H., Katz R., Cao J.J., Fried L.F., Kestenbaum B., Mukamal K., Rifkin D.E., Sarnak M.J., Shlipak M.G., Siscovick D.S. (2009). Cystatin C, albuminuria, and mortality among older adults with diabetes. Diabetes Care.

[54] Menard J., Campbell D.J., Azizi M., Gonzales M.F. (1997). Synergistic effects of ACE inhibition and Ang II antagonism on blood presure, cardiac weigt and renin in spontaneously hypertensive rats. Circulation.

[55] Jacobsen P., Andersen S., Rossing K., Jensen B.R., Parving H.H. (2003). Dual blockade of the renin-angiotensin system *versus* maximal recommended dose of ACE inhibition in diabetic nephropathy. Kidney Int..

[56] Jacobsen P., Andersen S., Jensen B.R., Parking H.H. (2003). Additive effect of ACE inhibition and angiotensin II receptor blockade in type I diabetic patients with diabetic nephropathy. J. Am. Soc. Nephrol..

[57] Mogensen C.E., Neldam S., Tikkanen I., Oren S., Viskoper R., Watts R.W., Cooper M.E. (2000). Randomised controlled trial of dual blockade of renin-angiotensin system in patients with hypertension, microalbuminuria, and non-insulin dependent diabetes: The Candesartan and Lisinopril Microalbuminuria (CALM) study. Br. Med. J..

[58] Bakris G.L., Ruilope L., Locatelli F., Ptaszynska A., Pieske B., de Champlain J., Weber M.A., Raz I. (2007). Treatment of microalbuminuria in hypertensive subjects with elevated cardiovascular risk: Results of the IMPROVE trial. Kidney Int..

[59] Kunz R., Friedrich C., Wolbers M., Mann J.F. (2008). Meta-analysis: Effect of monotherapy and combination therapy with inhibitors of the renin angiotensin system on proteinuria in renal disease. Ann. Intern. Med..

[60] Mancia G., de Backer G., Dominiczak A., Cifkova R., Fagard R., Germano G., Grassi G., Heagerty A.M., Kjeldsen S.E., Laurent S. (2007). 2007 ESH-ESC practice guidelines for the management of arterial hypertension. J. Hypertens..

[61] Yusuf S., Teo K.K., Pogue J., Dyal L., Copland I., Schumacher H., Dagenais G., Sleight P., Anderson C., ONTARGET Investigators (2008). Telmisartan, ramipril, or both in patients at high risk for vascular events. N. Engl. J. Med..

[62] Mann J.F., Schmieder R.E., McQueen M., Dyal L., Schumacher H., Pogue J., Wang X., Maggioni A., Budaj A., Chaithiraphan S., Dickstein K. (2008). ONTARGET investigators Renal outcomes with telmisartan, ramipril, or both, in people at high vascular risk (the ONTARGET study): A multicentre, randomised, double-blind, controlled trial. Lancet.

[63] Fried L.F., Duckworth W., Zhang J.H., O’Connor T., Brophy M., Emanuele N., Huang G.D., McCullough P.A., Palesvky P.M., Seliger S. (2009). Design of combination angiotensin receptor blocker and angiotensin-converting enzyme inhibitor for treatment of diabetic nephropathy (VA NEPHRON-D). Clin. J. Am. Soc. Nephrol..

[64] Imai E., Haneda M., Yamasaki T., Kobayashi F., Harada A., Ito S., Chan J.C., Makino H. (2013). Effects of dual blockade of the renin-angiotensin system on renal and cardiovascular outcomes in type 2 diabetes with overt nephropathy and hypertension in the ORIENT: A *post-hoc* analysis (ORIENT-Hypertension). Hypertens. Res..

[65] Persson F., Rossing P., Schjoedt K.J., Juhl T., Tarnow L., Stehouwer C.D.A., Schalkwijk C., Boomsma F., Frandsen E., Parving H.H. (2008). Time course of the antiproteinuric and antihypertensive effects of direct renin inhibition in type 2 diabetes. Kidney Int..

[66] Parving H.H., Persson F., Lewis J.B., Lewis E.J., Hollenberg N.K., AVOID Study Investigators (2008). Aliskiren combined with losartan in type 2 diabetes and nephropathy. N. Engl. J. Med..

[67] Parving H.H., Brenner B.M., McMurray J.J., de Zeeuw D., Haffner S.M., Solomon S.D., Chaturvedi N., Persson F., Desai A.S., Nicolaides M., Richard A. (2012). ALTITUDE Investigators Cardiorenal end points in a trial of aliskiren for type 2 diabetes. N. Engl. J. Med..

[68] Harel Z., Gilbert C., Wald R., Bell C., Perl J., Juurlink D., Beyene J., Shah P.S. (2012). The effect of combination treatment with aliskiren and blockers of the renin-angiotensin system on hyperkalaemia and acute kidney injury: Systematic review and meta-analysis. Br. Med. J..

[69] Bakris G.L., Oparil S., Purkayastha D., Yadao A.M., Alessi T., Sowers J.R. (2013). Randomized study of antihypertensive efficacy and safety of combination aliskiren/valsartan *vs.* valsartan monotherapy in hypertensive participants with type 2 diabetes mellitus. J. Clin. Hypertens. (Greenwich).

[70] Waanders F., Visser F.W., Gans R.O. (2013). Current concepts in the management of diabetic nephropathy. Neth. J. Med..

[71] Bomback A.S., Kshirsagar A.V., Amamoo M.A., Klemmer P.J. (2008). Change in proteinuria after adding aldosterone blockers to ACE inhibitors or angiotensin receptor blockers in CKD: A systematic review. Am. J. Kidney Dis..

[72] Sato A., Hayashi K., Naruse M., Saruta T. (2003). Effectiveness of aldosterone blockade in patients with diabetic nephropathy. Hypertension.

[73] Rachmani R., Slavachevsky I., Amit M., Levi Z., Kedar Y., Berla M., Ravid M. (2004). The effect of spironolactone, cilazapril and their combination on albuminuria in patients with hypertension and diabetic nephropathy is independent of blood pressure reduction: A randomized controlled study. Diabet. Med..

[74] Mehdi U.F., Adams-Huet B., Raskin P., Vega G.L., Toto R.D. (2009). Addition of angiotensin receptor blockade or mineralocorticoid antagonism to maximal angiotensin-converting enzyme inhibition in diabetic nephropathy. J. Am. Soc. Nephrol..

[75] Morales E., Millet V.G., Rojas-Rivera J., Huerta A., Gutiérrez E., Gutiérrez-Solís E., Egido J., Praga M. (2013). Renoprotective effects of mineralocorticoid receptor blockers in patients with proteinuric kidney diseases. Nephrol. Dial. Transplant..

[76] Epstein M., Williams G.H., Weinberger M., Lewin A., Krause S., Mukherjee R., Patni R., Beckerman B. (2006). Selective aldosterone blockade with eplerenone reduces albuminuria in patients with type 2 diabetes. Clin. J. Am. Soc. Nephrol..

[77] Mavrakanas T.A., Gariani K., Martin P.Y. (2014). Mineralocorticoid receptor blockade in addition to angiotensin converting enzyme inhibitor or angiotensin II receptor blocker treatment: An emerging paradigm in diabetic nephropathy: A systematic review. Eur. J. Intern. Med..

[78] Bakris G.L., Siomos M., Richardson D., Janssen I., Bolton W.K., Hebert L., Agarwal R., Catanzaro D., VAL-K Study Group (2000). ACE inhibition or angiotensin receptor blockade: Impact on potassium in renal failure. Kidney Int..

[79] Persson F., Rossing P. (2014). Sequential RAAS blockade: Is it worth the risk?. Adv. Chronic Kidney Dis..

[80] Bakris G.L., Barnhill B.W., Sadler R. (1992). Treatment of arterial hypertension in diabetic humans: Importance of therapeutic selection. Kidney Int..

[81] Böhlen L., de Courten M., Weidmann P. (1994). Comparative study of the effect of ACE-inhibitors and other antihypertensive agents on proteinuria in diabetic patients. Am. J. Hypertens..

[82] Bakris G.L., Copley J.B., Vicknair N., Sadler R., Leurgans S. (1996). Calcium channel blockers *versus* other antihypertensive therapies on progression of NIDDM associated nephropathy. Kidney Int..

[83] Bakris G.L., Weir M.R., DeQuattro V., McMahon F.G. (1998). Effects of an ACE inhibitor/calcium antagonist combination on proteinuria in diabetic nephropathy. Kidney Int..

[84] Vogt L., Waanders F., Boomsma F., de Zeeuw D., Navis G. (2008). Effects of dietary sodium and hydrochlorothiazide on the antiproteinuric efficacy of losartan. J. Am. Soc. Nephrol..

[85] Houlihan C.A., Allen T.J., Baxter A.L., Panangiotopoulos S., Casley D.J., Cooper M.E., Jerums G. (2002). A low-sodium diet potentiates the effects of losartan in type 2 diabetes. Diabetes Care.

[86] Slagman M.C., Waanders F., Hemmelder M.H., Woittiez A.J., Janssen W.M., Lambers Heerspink H.J., Navis G., Laverman G.D., HOlland NEphrology STudy Group (2011). Moderate dietary sodium restriction added to angiotensin converting enzyme inhibition compared with dual blockade in lowering proteinuria and blood pressure: Randomised controlled trial. BMJ.

[87] Lambers Heerspink H.J., Holtkamp F.A., Parving H.H., Navis G.J., Lewis J.B., Ritz E., de Graeff P.A., de Zeeuw D. (2012). Moderation of dietary sodium potentiates the renal and cardiovascular protective effects of angiotensin receptor blockers. Kidney Int..

[88] DiNicolantonio J.J., Pasquale P.D., Taylor R.S., Hackam D.G. (2013). Low sodium *versus* normal sodium diets in systolic heart failure: Systematic review and meta-analysis. Heart.

[89] Esnault V.L., Ekhlas A., Delcroix C., Moutel M.G., Nguyen J.M. (2005). Diuretic and enhanced sodium restriction results in improved antiproteinuric response to RAS blocking agents. J. Am. Soc. Nephrol..

[90] Collins A.J., Foley R.N., Chavers B., Gilbertson D., Herzog C., Ishani A., Johansen K., Kasiske B.L., Kutner N., Liu J. (2013). USRDS 2013 Annual Data Report: Atlas of Chronic Kidney Disease and End-Stage Renal Disease in the United States.

[91] Hsu T.W., Liu J.S., Hung S.C., Kuo K.L., Chang Y.K., Chen Y.C., Hsu C.C., Tarng D.C. (2014). Renoprotective effect of reninangiotensin-aldosterone system blockade in patients with predialysis advanced chronic kidney disease, hypertension, and anemia. JAMA Intern. Med..

[92] Yee J. (2014). Diabetic Kidney Disease: An ACEI (or an ARB) in the Hole. Adv. Chronic Kidney Dis..

[93] Apperloo A.J., de Zeeuw D., de Jong P.E. (1997). A short-term antihypertensive treatment-induced fall in glomerular filtration rate predicts long-term stability of renal function. Kidney Int..

[94] Holtkamp F.A., de Zeeuw D., Thomas M.C., Cooper M.E., de Graeff P.A., Hillege H.J., Parving H.H., Brenner B.M., Shahinfar S., Lambers Heerspink H.J. (2011). An acute fall in estimated glomerular filtration rate during treatment with losartan predicts a slower decrease in long-term renal function. Kidney Int..

[95] Hansen H.P., Rossing P., Tarnow L., Nielsen F.S., Jensen B.R., Parving H.H. (1995). Increased glomerular filtration rate after withdrawal of long-term antihypertensive treatment in diabetic nephropathy. Kidney Int..

[96] Onuigbo M.A., Onuigbo N.T. (2008). Worsening renal failure in older chronic kidney disease patients with renal artery stenosis concurrently on renin angiotensin aldosterone system blockade: A prospective 50-month Mayo-Health-System clinic analysis. QJM.

[97] Onuigbo M.A. (2008). Radiographic contrast-induced nephropathy and patient mortality. Mayo Clin. Proc..

[98] Onuigbo M.A. (2010). Syndrome of rapid-onset end-stage renal disease: A new unrecognized pattern of CKD progression to ESRD. Ren. Fail..

[99] Onuigbo M.A. (2013). Evidence of the syndrome of rapid onset end-stage renal disease (SORO-ESRD) in the acute kidney injury (AKI) literature—Preventable causes of AKI and SORO-ESRD—A call for re-engineering of nephrology practice paradigms. Ren. Fail..

[100] Bjorck S., Mulec H., Johnsen S.A., Norden G., Aurell M. (1992). Renal protective effect of enalapril in diabetic nephropathy. BMJ.

[101] (1998). Efficacy of atenolol and captopril in reducing risk of macrovascular and microvascular complications in type 2 diabetes: UKPDS 39. BMJ.

[102] Gerstein H.C., Miller M.E., Byington R.P., Goff D.C., Bigger J.T., Buse J.B., Cushman W.C., Genuth S., Ismail-Beigi F., Grimm R.H., Action to Control Cardiovascular Risk in Diabetes Study Group (2008). Effects of intensive glucose lowering in type 2 diabetes. N. Engl. J. Med..

[103] Cushman W.C., Evans G.W., Byington R.P., Goff D.C., Grimm R.H., Cutler J.A., Simons-Morton D.G., Basile J.N., Corson M.A., Probstfield J.L., The ACCORD Study Group (2010). Effects of intensive blood-pressure control in type 2 diabetes mellitus. N. Engl. J. Med..

[104] James P.A., Oparil S., Carter B.L., Cushman W.C., Dennison-Himmelfarb C., Handler J., Lackland D.T., LeFevre M.L., MacKenzie T.D., Ogedegbe O. (2014). 2014 evidence-based guideline for the management of high blood pressure in adults: Report from the panel members appointed to the eighth joint national committee (JNC 8). JAMA.

[105] Khosla N., Kalaitzidis R., Bakris G.L. (2009). Predictors of hyperkalemia risk following hypertension control with aldosterone blockade. Am. J. Nephrol..

[106] Van Buren P.N., Adams-Huet B. (2014). Potassium handling with dual renin-angiotensin system inhibition in diabetic nephropathy. Clin. J. Am. Soc. Nephrol..

[107] Eijkelkamp W.B., Zhang Z., Remuzzi G., Parving H.H., Cooper M.E., Keane W.F., Shahinfar S., Gleim G.W., Weir M.R., Brenner B.M. (2007). Albuminuria is a target for renoprotective therapy independent from blood pressure in patients with type 2 diabetic nephropathy: *Post hoc* analysis from the Reduction of Endpoints in NIDDM with the Angiotensin II Antagonist Losartan (RENAAL) trial. J. Am. Soc. Nephrol..

[108] Hou F.F., Xie D., Zhang X., Chen P.Y., Zhang W.R., Liang M., Guo Z.J., Jiang J.P. (2007). Renoprotection of Optimal Antiproteinuric Doses (ROAD) Study: A randomized controlled study of benazepril and losartan in chronic renal insufficiency. J. Am. Soc. Nephrol..

[109] Rossing K., Schjoedt K.J., Jensen B.R., Boomsma F., Parving H.H. (2005). Enhanced renoprotective effects of ultrahigh doses of irbesartan in patients with type 2 diabetes and microalbuminuria. Kidney Int..

[110] Burgess E., Muirhead N., Rene de Cotret P., Chiu A., Pichette V., Tobe S., SMART (Supra Maximal Atacand Renal Trial) Investigators (2009). Supramaximal dose of candesartan in proteinuric renal disease. J. Am. Soc. Nephrol..

[111] Wheeler D.C., Becker G.J. (2013). Summary of KDIGO guideline. What do we really know about management of blood pressure in patients with chronic kidney disease?. Kidney Int..

[112] Taler S.J., Agarwal R., Bakris G.L., Flynn J.T., Nilsson P.M., Rahman M., Sanders P.W., Textor S.C., Weir M.R., Townsend R.R. (2013). KDOQI US commentary on the 2012 KDIGO clinical practice guideline for management of blood pressure in CKD. Am. J. Kidney Dis..

[113] Yamout H., Lazich I., Bakris G.L. (2014). Blood pressure, hypertension, RAAS blockade, and drug therapy in diabetic kidney disease. Adv. Chronic Kidney Dis..

[114] Wong J., Molyneaux L., Constantino M., Twigg S.M., Yue U.K. (2010). Beyond ONTARGET: Angiotensin-converting enzyme inhibition and angiotensin II receptor blockade in combination, a lesser evil in some?. Diabetes Obes. Metab..

